# Outcome and prognostic factors of patients with right-sided infective endocarditis requiring intensive care unit admission

**DOI:** 10.1186/s12879-018-2989-9

**Published:** 2018-02-21

**Authors:** Hugues Georges, Olivier Leroy, Norair Airapetian, Nicolas Lamblin, Elie Zogheib, Patrick Devos, Sebastien Preau, Hauts de France endocarditis study group

**Affiliations:** 1 Service de Réanimation Médicale et Maladies Infectieuses, Hôpital Chatiliez, 135 rue du Président, 59200 Tourcoing, France; 2 Réanimation polyvalente, CHU Amiens Picardie, Amiens, France; 3 Pôle de cardiologie, Hôpital cardio-thoracique, CHU Lille, Avenue du Professeur E. Laine, 59037 Lille Cedex, France; 4 Université LILLE. EA 2694 - Santé publique : Epidémiologie et qualité des soins, CHU de Lille, 59000 Lille, France; 5 Pôle de réanimation, Hôpital R. Salengro, CHU de Lille, Avenue du Professeur E. Laine, 59000 Lille Cedex, France

**Keywords:** Right-sided infective endocarditis, Intensive care unit, Prognostic factors

## Abstract

**Background:**

Right-sided infective endocarditis (RSIE) is an uncommon diagnosis accounting for less than 10% of cases of infective endocarditis. Optimal management for severely ill patients with RSIE remains challenging because few studies reported on management and outcome. The goal of our study was to determine outcome and associated prognostic factors in a population of ICU patients with a diagnosis of definite, active and severe RSIE.

**Methods:**

We performed a retrospective study in 10 French ICUs between January 2002 and December 2012. Main outcome was mortality at 30 days after ICU admission. Significant variables associated with 30-days mortality in the bivariate analysis were included in a logistic regression analysis.

**Results:**

A total of 37 patients were studied. Mean age was 47.9 ± 18.4 years. Mean SAPS II, SOFA score and Charlson comorbidity index were 32.4 ± 17.4, 6.3 ± 4.4 and 3.1 ± 3.4, respectively. Causative pathogens, identified in 34 patients, were mainly staphylococci (*n* = 29). The source of endocarditis was a catheter related infection in 10 patients, intravenous drug abuse in 8 patients, cutaneous in 7 patients, urinary tract related in one patient and has an unknown origin in 7 patients. Vegetation size was higher than 20 mm for 14 patients. Valve tricuspid regurgitation was classified as severe in 11 patients. All patients received initial appropriate antimicrobial therapy. Aminoglycosides were delivered in combination with β-lactam antibiotics or vancomycin in 22 patients. Surgical procedure was performed in 14 patients. Eight patients (21.6%) died within 30 days following ICU admission. One independent prognostic factor was identified: use of aminoglycosides was associated with improved outcome (OR = 0.1; 95%CI = 0.0017–0.650; *p* = 0.007).

**Conclusion:**

Mortality of patients with RSIE needing ICU admission is high. Aminoglycosides used in combination with β-lactam or vancomycin could reduce 30 days mortality.

## Background

Right-sided infected endocarditis (RSIE) account for 5–10% of all episodes of endocarditis [1, 2]. RSIE have been essentially described in injecting drug users (IDUs), but other causes as catheter related infections (CRI) or skin lesions have been reported [3–5]. Increasing use of implantable cardiac electronic device (ICED), peripherally inserted central catheter (PICC lines), and subcutaneous implanted “PORT” catheter are also practices leading to the development of RSIE in the future [6, 7].

Morbidity and mortality of RSIE are generally reported as less severe than left-sided endocarditis with a mortality rate ranging from 3 to 30% according to the source of infection [8, 9]. RSIE is a rare cause of ICU admission because of the low incidence and the relative severity of this disease. Studies relating prognostic factors and outcome of RSIE in patients requiring ICU admission are thus rare. Most often, they described specific type of population like IDUs or general population of patients with infective endocarditis without specific analysis in RSIE patients [10–12].

To our knowledge no study has reported epidemiology, prognostic factors and outcome of ICU patients with RSIE and diverse source of infection. Also, therapeutic management proposed by international guidelines including antimicrobial therapy and surgical treatment of RSIE have not been validated in more severely ill patients [13, 14].

Therefore, we conducted a multicentre retrospective analysis of critically ill patients with RSIE in order to evaluate first outcome and prognosis factors and secondly relevance of therapeutic procedures proposed by the last internationals guidelines.

## Methods

### Study design and patients

We performed this multicentre retrospective study in 10 ICUs of 8 hospitals (Amiens, Boulogne sur Mer, Douai, Dunkerque, Valenciennes, Lille, Roubaix, and Tourcoing) in the north of France, between January 2002 and December 2012. Amiens and Lille hospitals were both academic hospitals with cardiac surgery department. All other hospitals were general hospitals.

Adult patients were included in the study if they had a right-sided, definitive, active and severe infective endocarditis (IE) requiring ICU admission. IE was defined according to modified Duke Criteria [15]. As all these criteria could not be present on ICU admission, there may be a delay between ICU admission and IE diagnosis. Endocarditis was defined as active if the patient was admitted in ICU before or within the 30 days of antimicrobial treatment. IE was considered as severe when associated with any of the following criteria: acute respiratory failure requiring mechanical ventilation, shock, Simplified Acute Physiology Score (SAPS II) ≥ 20 and Sequential Organ Failure Assessment (SOFA) ≥ 3 [16, 17].

Patients with both right and left side IE were not included in the study as well as patients with ICED infection without tricuspid or pulmonary sided valve vegetation.

### Collected data and definitions

For all patients, information was collected from medical records, anonymously entered into a database and reviewed for data entry errors and/or inconsistencies.

For each patient, data about age, gender, cause of ICU admission, severity of illness, pre-existing comorbidities, source of infection, time of diagnosis (before or during ICU admission)*,* acquisition of endocarditis during ICU stay, presence of associated ICED, presence of prosthetic tricuspid valve, type of endocarditis, echocardiographic data, occurrence of neurological complications or septic pulmonary embolism, microbiological identification and therapeutic management were collected.

- Cause of ICU admission was classified according to main diagnosis: septic shock, cardiac failure, respiratory failure, renal failure or other.
- Severity of illness was assessed by the SOFA score and SAPS II on ICU admission [16, 17].
- Pre-existing comorbidities were evaluated using the Charlson Comorbidity Index [18].
- Source of infection was classified as following: cutaneous, IDUs, CRI, other origin or unknown. CRI included long term catheter (Port-a-cath, PICC-Line), central venous catheter and peripheral venous catheter.
- Echocardiographic data included vegetation size, severity of tricuspid regurgitation, left ventricular ejection fraction (LVEF), infection of associated ICED. Tricuspid regurgitation was defined as severe using current definition [19].
- Type of IE was classified as community-acquired, nosocomial or ICED-associated. RSIE was defined as nosocomial if endocarditis occurred more than 72 h after admission to the hospital. Patients were classified as ICED-associated IE when infection of a pacemaker or implantable cardiac defibrillators was determined. IE was defined as acquired during ICU stay if reason for ICU admission was not IE or organ failure related to IE
- Septic pulmonary embolism was diagnosed by computed tomography.
- Microbial diagnosis was assessed according to the modified Duke criteria [15].
- Collected data concerning therapeutic management included use of aminoglycosides, surgical treatment and removal of ICED. The guidelines recommendations concerning delivery of aminoglycosides and indications for surgical procedure were then retrospectively studied [13]. For the needs of this evaluation**,** use of aminoglycosides was defined as guideline-concordant if (1) the treatment was delivered in the clinical situations defined by the guideline (2) the treatment was not delivered in the absence of indication. Indication for surgery was determined by the physicians in charge of the patient and was assessed according to the three recommendations supported by international guidelines: difficulty for microbial eradication or persistent bacteraemia, right heart failure (RHF) with severe tricuspid regurgitation, diameter of valve vegetation higher than 20 mm after recurrent pulmonary embolism [13, 14]. For the study, surgery procedure was classified as infectious, hemodynamic, embolic or combination of them.

### Outcome definition

Main outcome measure was 30-day mortality following ICU admission.

### Statistical analysis

Two groups of patients were defined: Survivors and non survivors at 30 days following ICU admission. Data from the two studied groups were compared using the chi-squared test or Fischer exact test for categorical parameters and student’s *t* test for continuous variables. Quantitative variables are reported as mean ± standard deviation. Qualitative variables are reported as number and percentage. When appropriate, continuous variables were analyzed as categorical variables using clinically meaningful cut-off points*.* We performed a bivariate analysis including all patients to assess risk factors for mortality. Difference between the two studied groups was considered to be significant for variables yielding a *p* value ≤0.05. Then a stepwise logistic regression analysis was performed, including variables with a p value ≤0.10 in the bivariate analysis, to identify independent risk factors associated with mortality. Adjusted odds ratios were computed using logistic regression analysis including the independent predictors of mortality. All statistical analyses were performed using SAS software, version 9.1 (SAS Institute, Inc., Cary, NC).

## Results

### Characteristics of the studied population

A total of 37 patients were admitted in our ICUs during the study period. Mean age was 47.9 ± 18.4 years and 54% of patients were male gender. Causes of ICU admission were classified as following: respiratory failure in 12 patients, cardiac failure in 11 patients, septic shock in 9 patients, renal failure in 4 patients and other cause in one patient. Mean SAPS II, SOFA score and Charlson comorbidity index were 32.4 ± 17.4, 6.3 ± 4.4 and 3.1 ± 3.4, respectively. Endocarditis was classified as community-acquired in 19 patients, hospital-acquired in 14 patients and ICED-associated in 4 patients. Endocarditis was acquired in ICU for 3 patients with a diagnosis performed 10, 15 and 17 days following ICU admission. The source of endocarditis was a CRI in 10 patients, IDUs in 8 patients, cutaneous in 7 patients, urinary tract related in one patient and has an unknown origin in 7 patients. In patients with CRI, source of infection was central venous catheter in 5 patients, peripheral venous catheter in 2 patients and long term catheter in 3 patients. Thirty five patients had native valve infection and 2 patients had prosthetic valve infection. Endocarditis was diagnosed before ICU admission for 12 patients and was acquired in ICU for 3 patients. Pulmonary CT scan was performed in 30 patients and a septic pulmonary embolism was diagnosed (with RSIE) in 24 (64.9%) patients. A brain CT scan was performed in 19 patients and was pathological in 4 patients with lesions classified as ischemia. One patient presented with meningitis. All patients without CT scan had normal neurological examination. The median length of stay in ICU and hospital were 14.3 ± 15.6 and 42.5 ± 27 days, respectively.

### Echographic characteristics

One patient had a tricuspid valve perforation. All other patients presented with at least one vegetation. Vegetation size was determined in 32 patients. Mean maximum vegetation length was 19.8 ± 10.5 mm. Vegetation size was higher than 20 mm for 14 (43.7%) patients. Valve tricuspid regurgitation was classified as severe in 11 (29.7%) patients. LVEF was below 50% in 8 (21.6%) patients. ICED lead infection was diagnosed in four patients with tricuspid valve vegetation.

### Microbial diagnosis

Forty causative pathogens were identified in 34 patients (Table 1). The most common isolated pathogens were staphylococci (*n* = 29). *S. aureus* (*n* = 26) was identified in 25 patients, one patient having both methicillin-sensitive and methicillin-resistant *S. aureus* isolated on blood cultures.

**Table 1:** Causative pathogens isolated in 34 patients with microbial documentation 2 pathogens were identified for 6 patients

Pathogen	n (%)
*Staphylococcus aureus*	26 (65)
Methicillin-susceptible	17 (42.5)
Methicillin-resistant	9 (22.5)
Coagulase-negative *Staphylococcus*	3 (7.5)
Gram-negative bacilli	4 (10)
*Streptococcus* spp	1 (2.5)
*Candida* spp	5 (12.5)
*Enterococcus* spp	1 (2.5)

### Therapeutic management

All patients have received at least one appropriate antimicrobial agent within 48 h following the diagnosis of endocarditis. Use of aminoglycosides therapy could be evaluated in 34 patients. Among these, aminoglycosides were recommended in 32 (94.1%) patients and were delivered for only 22 (68.7%) of them. In the group of patients with *S. aureus* endocarditis (*n* = 25) aminoglycosides were recommended in 23 patients and were delivered for 17 (73.9%) of them. Two patients received aminoglycosides while there was no recommendation. Surgery was indicated for 14 (37.8%) patients with two indications in 5 patients. Indications were RHF in 8 patients and size of vegetation associated with pulmonary embolism in 11 patients. Among these 14 patients, 10 underwent cardiac surgery. Four patients have had surgical procedure while there was no theoretical indication in current guidelines.

Surgical procedure consisted in tricuspid valve replacement in 5 patients, vegectomy in 5 patients and tricuspid valve repair with vegectomy in 3 patients. No data concerning performed surgical procedure was found for one patient.

Removal of ICED was performed in two of four patients with endocarditis associated with ICED infections.

### Outcome and prognosis factors

Eight (21.6%) patients were dead at 30 days after ICU admission. The overall in-hospital mortality was 12 (32.4%) patients. No IDU died. One patient died among the fourteen operated. Among the 4 patients without surgery despite guidelines recommendation, three were alive within 30 days following ICU admission and discharged from hospital. Indications for surgery were respectively diameter of vegetation for one patient, right heart failure for one patient and these two associated reasons for one patient.

Bivariate analysis assessing prognosis factors is reported in Table 2. Main significant factors associated with 30 days survival were age lower than 45 years, Charlson score < 3, endocarditis diagnosed before ICU admission, aminoglycosides use, presence of septic pulmonary embolism and a single surgical indication for patients needing surgical procedure.

**Table 2:** Bivariate analysis of risk factors for mortality at 30 days following the diagnosis on ICU

Factor	Survivors n = 29	Non survivors *n* = 8	*p*
Gender, Male/Female	17/12	3/5	0.28
Mean Age	44.8 ± 16.5	67.2 ± 14.1	< 0.004
Age > 45 years	12 (41.3)	8 (100)	< 0.004
Cause of ICU admission			
Septic shock	7 (24.1)	2 (25)	= 0.9
Cardiac failure	9 (31)	2 (25)	= 0.7
Respiratory failure	11 (37.9)	1 (12.5)	= 0.18
Renal failure	2 (6.9)	2 (25)	= 0.14
Other causes	0 (0)	1 (12.5)	
Mean SAPS II	29.6 ± 14.8	42.5 ± 22.9	= 0.12
Mean SOFA	5.9 ± 4.2	7.6 ± 5.0	= 0.39
Charlson score	2.5 ± 3.3	4.8 ± 3.3	< 0.05
Charlson score > 3	10 (34.5)	6 (75)	< 0.05
Diagnosis before ICU admission	12 (41.3)	0 (0)	< 0.03
RSIE acquired in ICU	2 (6.9)	1(12.5)	= 0.6
Community-acquired RSIE	12 (41.4)	6 (75)	= 0.09
Nosocomial endocarditis	10 (34.5)	4 (50)	= 0.42
ICED associated endocarditis	2 (6.9)	2 (25)	= 0.14
Source of infection			
cutaneous	4 (13.8)	3 (37.5)	= 0.12
IVDA	8 (27.6)	0 (0)	= 0.09
Catheter related infection	9 (31)	1 (12.5)	= 0.28
unknown	6 (20.7)	1 (12.5)	= 0.6
other	0 (0)	1 (12.5)	= 0.05
Native tricuspid valve endocarditis	28 (96.6)	7 (87.5)	= 0.31
Severe tricuspid regurgitation	9 (31)	2 (25)	= 0.74
Vegetation size	19.0 **±** 9.7	23.0 **±** 13.9	= 0.59
LVEF < 50%	6 (20.7)	2 (25)	= 0.79
Neurological complications	3 (10.3)	2 (25)	< 0.02
Septic pulmonary embolism (*n* = 30)*	23/26 (88.6)	1/4 (25)	< 0.002
Pathogens			
Methicillin-sensible *S. aureus*	15 (51.7)	2 (25)	= 0.2
Methicillin-resistant *S. Aureus*	6 (20.7)	3 (37.5)	= 0.27
Fungal	2 (6.9)	2 (25)	= 0.12
Other pathogens	8 (27.6)	0 (0)	= 0.09
Aminoglycosides indication (*n* = 34)**	27/28	5/6	= 0.23
Aminoglycosides delivery (n = 34)**	22/28	2/6	< 0.05
Aminoglycosides conformity(*n* = 34)**	21/28	1/6	< 0.02
Surgical indication	12 (41.4)	2 (25)	= 0.39
Indication: size of vegetation	9 (31)	2 (25)	= 0.42
Indication: RHF	6 (20.7)	2 (25)	= 0.18
Two indications	3 (10.3)	2 (25)	< 0.05
Surgical procedure performed	13 (44.8)	1 (12.5)	= 0.09

Data are presented as No (%)

* CT scan has not been performed for seven patients

** No Evaluation could be made for 3 patients

Multivariate analysis identified only one prognostic factor: use of aminoglycosides was associated with improved outcome (OR = 0.1; 95% CI: 0.017–0.650; *p* = 0.007).

Among patients with *S. aureus* endocarditis, 5 patients died (Table 3). Mortality was associated with lack of aminoglycosides administration: among the 5 dead patients, only one received aminoglycoside while treatment was recommended for 4 of them. Use of aminoglycosides was lower in patients with methicillin-resistant *S. aureus* (MRSA) endocarditis (Table 4). The two patients with IE and not removal of infected ICED died.

**Table 3:** Outcome, indication, use and assessment of aminoglycosides in the group of patients with *S. aureus* endocarditis

	Survivors (*n* = 20)	Non survivors (*n* = 5)	*p*
Indication of aminoglycosides	19/20	4/5	= 0.26
Delivery of aminoglycosides	15/20	2/5	= 0.12
Use of aminoglycosides in Patients with indication	14/19 (73.6%)	1/4 (25%)	= 0.07
Aminoglycosides conformity	14/20 (70%)	1/5 (20%)	< 0.05

**Table 4:** Indication, use and assessment of aminoglycosides in patients with *S. aureus* IE (infective endocarditis) according to methicillin susceptibility

	IE due to MSSA (*n* = 17)	IE due to MRSA (*n* = 9)	*p*
Indication of aminoglycosides	15 (88.2)	9 (100)	= 0.28
Delivery of aminoglycosides	14 (82.3)	4 (44.4)	< 0.05
Use of aminoglycosides in patients with indication	12 (70.6)	4 (44.4)	= 0.18
Aminoglycosides conformity	12 (70.6)	4 (44.4)	= 0.18
Number of deaths	2 (11.8)	3 (33.3)	= 0.18

Data are presented as No (%)

*MSSA* methicillin-susceptible *S. aureus*

*MRSA* methicillin-resistant *S. aureus*

## Discussion

We report results of a retrospective multicenter study on 37 patients with severe, active, definite RSIE requiring ICU admission*.* Mortality at 30 days following ICU admission was 21.6% and the only independent prognosis factor decreasing mortality was the adequate use of aminoglycosides as recommended in the last published ESC guidelines.

Mortality of patients with RSIE varies according to origin of infection and preexisting comorbidities. Prognosis is generally favorable in young and otherwise healthy IDUs which represent the largest part of studied patients with a mortality rate less than 5–10% [8, 20]. These patients are generally described with minimal right-sided valve dysfunction, low risk of pulmonary embolism and good response to appropriate antibiotic therapy. The only study describing outcome of IDUs with RSIE needing ICU admission reported a mortality of 26% [10]. We did not find the same results since no IDU died in our study despite a similar severity. We have no explanation for this difference but antibiotics used and indications for surgical procedure were not specified in the study of Saydain and coll. Prognosis is generally more severe in non IDUs. Mortality was 30% in a study including patients without ICED or drug abuse [9]. Fernandez Guerrero and coll. Reported nine deaths in a population of 11 patients with RSIE caused by intravascular catheter infection [21]. We have not the same results because only one patient on nine with RSIE caused by catheter-related infection died. Systematic catheter removal in patients with staphylococcal bacteremia could explain this low mortality rate. In our study, mortality 30 days after ICU admission was 21.6% for the full studied population and 27.6% for the group including non intra venous drug abuse patients. Overall in-hospital mortality was 32.4%. Ours results were similar to the results of Mourvillier and coll reporting outcome in patients with infective endocarditis requiring ICU admission: among the 228 studied patients, 26 were admitted with RSIE without left-side IE [11]. Eight (31%) of the 26 patients died but no specific analysis was performed to assess prognosis factors.

In our report, the only prognosis factor associated with improved survival is the administration of aminoglycosides in combination with penicillins or vancomycin. Addition of gentamicin to oxacillin or vancomycin is a standard approach for the treatment of RSIE. This combination is associated with a good in vitro synergy against *S. aureus* rapidly leading to bactericidal activity [22]. A rabbit model confirms this result with a more rapid eradication of *S. aureus* from cardiac vegetation [22]. However, even if a study has showed that combination therapy results in a reduced duration of bacteremia in patients with *S. aureus* RSIE, uncertainty about the therapeutic advantage of aminoglycosides remains, supported by clinical studies relating lack of benefit in clinical course or significant worsening of renal dysfunction [23–25]. Recently, the 2015 American and European updates of guidelines no longer suggest using aminoglycosides in the treatment of native valve staphylococcal endocarditis, regardless of the sensitive or resistant nature of the strain to methicillin [13, 14]. However, an uncertainty remains about the delivery of aminoglycosides in patients with RSIE. If we follow recommendations published from the same guidelines, gentamicin must be used in the initial treatment in combination with betalactamin or glycopepetide and a two week treatment without gentamicin is proposed only in patients with uncomplicated methicillin-sensitive *S. Aureus* (MSSA) RSIE. We wanted to assess these recommendations in our studied patients. Aminoglycosides combination was then recommended in 23 patients with staphylococcal endocarditis and in 32 patients in all. Our results are in opposition with current recommendation concerning the antimicrobial regimen for staphylococcal endocarditis [26]. We showed the necessity to deliver aminoglycosides in complicated RSIE, including *S. aureus* RSIE. According to our results, use of aminoglycosides for a duration from 3 to 5 days is advised in patients with MRSA RSIE and in patients with complicated MSSA RSIE, defined by the presence of metastatic sites of infection, cardiac or extracardiac complications and length of vegetation higher than 20 mm.

Several prognosis factors have been described in other reports assessing outcome of RSIE patients. Vegetation length > 20 mm and fungal origin were the main predictors of death in a recent retrospective study [27]. High sepsis score defined by the Pittsburgh bacteremia score ≥ 4 was associated with a higher mortality in IDUs with RSIE [3]. In our study causative pathogens, size of vegetation and severity of sepsis were not prognostic factors. Many reasons could explain this result. First, we studied severely ill patients needing specific cares conducted by critical care physicians having a certain expertise in the delivery of antimicrobial agents and management of severe sepsis and septic shock. Second, a surgical referral unit was available for all participating hospitals with the possibility to discuss all surgical indications. However we found in bivariate analysis that underlying conditions defined by an age > 45 year and a Charlson score > 3 were associated with higher mortality.

Most patients with RSIE are treated with medical therapy alone and surgery concerns generally a minority of cases. Surgical treatment varies in different studies with a rate of operated patients ranging from 5 to 40% of cases [3, 9, 28]. If the occurrence of congestive cardiac failure is an established indication for surgery, the assertion that vegetation size should be a criterion for considering surgical intervention is based on a small number of studies with methodological flaws [2, 29]. In our study, surgical procedure has been performed for 14 patients. Two guidelines could serve as references for most patients during our studied period: ESC guidelines on Prevention, Diagnosis and Treatment of Infective Endocarditis and AHA Scientific statement on infective endocarditis published respectively in 2004 and 2005 [30, 31]. No clear surgical recommendations concerning RSIE were proposed in these two guidelines. In our study we have chosen to assess the recommendations published in 2015: surgical treatment should be considered in patients with right HF secondary to severe tricuspid regurgitation with poor response to diuretic therapy, patients with IE caused by organism which are difficult to eradicate or duration of bacteremia for at least 7 days and patients with tricuspid valve vegetation > 20 mm which persists after recurrent pulmonary emboli [13, 14]. These criteria concerned 14 patients (37.8%) in our study. This rate is higher than described in literature and reflects probably the disease severity of our patients. Among these, 10 were operated and one patient died. Among the 4 patients not operated despite guidelines recommendations, 3 were discharged alive from ICU and hospital. One patient had two surgical indications and two patients had only one surgical indication, right heart failure and length of vegetation. The dead patient had two surgical indications. Other studies are thus required to validate surgical recommendations in ICU patients.

Our results confirm the absolute necessity to remove ICED associated endocarditis. In our study both patients without removal of infected ICED are dead. Recently a guideline relating to the management of ICED infection recommends the complete and early removal of all infected ICED system combined with appropriate antimicrobial therapy as a safe and efficient treatment option [6].

Our study presents some limitations. First, all data were collected retrospectively in a multicenter setting. Thus, diagnostic methods and therapeutic measures were not standardized.

Second, the studied period was of 11 years and therapeutic measures, like management of sepsis and its complications can have changed during this long period of time. Third, we have included few patients and results of our study need to be confirmed in further studies. Fourth, other important guidelines recommendations have not been approached as the choice and appropriateness of antimicrobial therapy according to underlying conditions. Diagnosis of RSIE was principally made after receiving results of blood cultures thus reducing the risk of inadequate delivery of antimicrobial therapy. Fifth, we have not assessed occurrence of nephrotoxicity during treatment with the use of aminoglycosides.

## Conclusion

In conclusion, mortality of patients with RSIE needing ICU admission is high. *S aureus* is the main causative pathogen and must always be covered by initial antimicrobial therapy. Our results suggest that aminoglycosides used in combination with β-lactam or vancomycin could reduce 30 days mortality.

## Abbreviations

CRI: catheter related infection
ESC: European society of cardiology
ICED: implantable cardiac electronic device
ICU: Intensive care unit
IDUs: Injecting drug users
IE: Infective endocarditis
LVEF: left ventricular ejection fraction
MRSA: methicillin-resistant *Staphylococcus aureus*
MSSA: methicillin-susceptible *Staphylococcus aureus*
PICC: peripherally inserted central catheter
RSIE: Right-sided infective endocarditis
SAPS II: Simplified acute physiologic score
SOFA: Sequential Organ Failure Assessment

## References

[1] Chan P, Ogilby J, Segal B (1989). Tricuspid valve endocarditis. Am Heart J.

[2] Murdoch DR, Corey GR, Hoen B, Miró JM, Fowler VG, Bayer AS (2009). International collaboration on endocarditis-prospective cohort study (ICE-PCS) investigators. Clinical presentation, etiology, and outcome of infective endocarditis in the 21st century: the international collaboration on endocarditis-prospective cohort study. Arch Intern Med.

[3] Otome O, Guy S, Tramontana A, Lane G, Karunajeewa H (2016). A retrospective review: significance of vegetation size in injection drug users with right-sided infective endocarditis. Heart Lung Circ.

[4] Yamashita S, Noma K, Kuwata G, Miyoshi K, Honaga K (2005). Infective endocarditis at the tricuspid valve following central venous catheterization. J Anesth.

[5] Armstrong ML, DeBoer S, Cetta F (2008). Infective endocarditis after body art: a review of the literature and concerns. J Adolesc Health.

[6] Sandoe JA, Barlow G, Chambers JB, Gammage M, Guleri A, Howard P (2015). British Society for Antimicrobial Chemotherapy; British Heart Rhythm Society; British cardiovascular society; British heart valve society; British Society for Echocardiography. Guidelines for the diagnosis, prevention and management of implantable cardiac electronic device infection. Report of a joint working party project on behalf of the British Society for Antimicrobial Chemotherapy (BSAC, host organization), British Heart Rhythm Society (BHRS), British cardiovascular society (BCS), British heart valve society (BHVS) and British Society for Echocardiography (BSE). J Antimicrob Chemother.

[7] Hussain ST, Witten J, Shrestha NK, Blackstone EH, Pettersson GB (2017). Tricuspid valve endocarditis. Ann Cardiothorac Surg.

[8] Moss R, Munt B (2003). Injection drug use and right sided endocarditis. Heart.

[9] Ortiz C, López J, García H, Sevilla T, Revilla A, Vilacosta I (2014). Clinical classification and prognosis of isolated right-sided infective endocarditis. Medicine (Baltimore).

[10] Saydain G, Singh J, Dalal B, Yoo W, Levine DP (2010). Outcome of patients with injection drug use-associated endocarditis admitted to an intensive care unit. J Crit Care.

[11] Mourvillier B, Trouillet JL, Timsit JF, Baudot J, Chastre J, Régnier B (2004). Infective endocarditis in the intensive care unit: clinical spectrum and prognostic factors in 228 consecutive patients. Intensive Care Med.

[12] Samol A, Kaese S, Bloch J, Görlich D, Peters G, Waltenberger J (2015). Infective endocarditis on ICU: risk factors, outcome and long-term follow-up. Infection.

[13] Habib G, Lancellotti P, Antunes MJ, Bongiorni MG, Casalta JP, Del Zotti F (2015). 2015 ESC guidelines for the Management of Infective Endocarditis: the task force for the management of infective endocarditis of the European Society of Cardiology (ESC). Endorsed by: European Association for Cardio-Thoracic Surgery (EACTS), the European Association of Nuclear Medicine (EANM). Eur Heart J.

[14] Baddour LM, Wilson WR, Bayer AS, Fowler VG Jr, Tleyjeh IM, Rybak MJ, et al. American Heart Association Committee on rheumatic fever, endocarditis, and Kawasaki disease of the council on cardiovascular disease in the young, council on clinical cardiology, council on cardiovascular surgery and anesthesia, and stroke council. Infective endocarditis in adults: diagnosis, antimicrobial therapy, and Management of Complications: a scientific statement for healthcare professionals from the American Heart Association. Circulation 2015;132(15):1435–1486. 10.1161/CIR.0000000000000296 26373316

[15] Li JS, Sexton DJ, Mick N, Nettles R, Fowler VG, Ryan T (2000). Proposed modifications to the Duke criteria for the diagnosis of infective endocarditis. Clin Infect Dis.

[16] Le Gall JR, Lemeshow S, Saulnier F (1993). A new simplified acute physiology score (SAPS II) based on a European/north American multicenter study. JAMA.

[17] Vincent JL, Moreno R, Takala J, Willatts S, De Mendonça A, Bruining H (1996). The SOFA (sepsis-related organ failure assessment) score to describe organ dysfunction/failure. On behalf of the working group on sepsis-related problems of the European Society of Intensive Care Medicine. Intensive Care Med.

[18] Charlson ME, Pompei P, Ales KL, MacKenzie CR (1987). A new method of classifying prognostic comorbidity in longitudinal studies: development and validation. J Chronic Dis.

[19] Zoghbi WA, Enriquez-Sarano M, Foster E, Grayburn PA, Kraft CD, Levine RA (2003). American Society of Echocardiography. J Am Soc Echocardiogr.

[20] Miró JM, del Río A, Mestres CA (2003). Infective endocarditis and cardiac surgery in intravenous drug abusers and HIV-1 infected patients. Cardiol Clin.

[21] Fernández Guerrero ML, González López JJ, Goyenechea A, Fraile J, de Górgolas M (2009). Endocarditis caused by Staphylococcus Aureus: a reappraisal of the epidemiologic, clinical, and pathologic manifestations with analysis of factors determining outcome. Medicine (Baltimore).

[22] Sande MA, Nafcillin-gentamicin CKB (1976). Synergism in experimental staphylococcal endocarditis. J Lab Clin Med.

[23] Korzeniowski O, Sande MA (1982). Combination antimicrobial therapy for Staphylococcus Aureus endocarditis in patients addicted to parenteral drugs and in nonaddicts: a prospective study. Ann Intern Med.

[24] Ribera E, Gómez-Jimenez J, Cortes E, del Valle O, Planes A, Gonzalez-Alujas T (1996). Effectiveness of cloxacillin with and without gentamicin in short-term therapy for right-sided Staphylococcus Aureus endocarditis. A randomized, controlled trial. Ann Intern Med.

[25] Cosgrove SE, Vigliani GA, Fowler VG Jr, Abrutyn E, Corey GR, Levine DP, et al. Initial low-dose gentamicin for Staphylococcus Aureus bacteremia and endocarditis is nephrotoxic. Clin Infect Dis 2009;48(6):713–721. 10.1086/597031 19207079

[26] Tattevin P, Mainardi JL (2016). Analysis of the 2015 American and European guidelines for the management of infective endocarditis. Med Mal Infect.

[27] Martín-Dávila P, Navas E, Fortún J, Moya JL, Cobo J, Pintado V (2005). Analysis of mortality and risk factors associated with native valve endocarditis in drug users: the importance of vegetation size. Am Heart J.

[28] Musci M, Siniawski H, Pasic M, Grauhan O, Weng Y, Meyer R (2007). Surgical treatment of right-sided active infective endocarditis with or without involvement of the left heart: 20-year single center experience. Eur J Cardiothorac Surg.

[29] Revilla A, López J, Villacorta E, Gómez I, Sevilla T, del Pozo MA, de la Fuente L (2008). Isolated right-sided valvular endocarditis in non-intravenous drug users. Rev Esp Cardiol.

[30] Horstkotte D, Follath F, Gutschik E, Lengyel M, Oto A, Pavie A (2004). Task force members on infective endocarditis of the European Society of Cardiology; ESC Committee for practice guidelines (CPG); document reviewers. Guidelines on prevention, diagnosis and treatment of infective endocarditis executive summary; the task force on infective endocarditis of the European society of cardiology. Eur Heart J.

[31] Baddour LM, Wilson WR, Bayer AS, Fowler VG Jr, Bolger AF, Levison ME, et al; Committee on Rheumatic Fever, Endocarditis, and Kawasaki Disease; Council on Cardiovascular Disease in the Young; Councils on Clinical Cardiology, Stroke, and Cardiovascular Surgery and Anesthesia; American Heart Association; Infectious Diseases Society of America. Infective endocarditis: diagnosis, antimicrobial therapy, and management of complications: a statement for healthcare professionals from the Committee on Rheumatic Fever, Endocarditis, and Kawasaki Disease, Council on Cardiovascular Disease in the Young, and the Councils on Clinical Cardiology, Stroke, and Cardiovascular Surgery and Anesthesia, American Heart Association: endorsed by the Infectious Diseases Society of America. Circulation. 2005;111(23):e394–434. 10.1161/CIRCULATIONAHA.105.165564 15956145

